# A comparative analysis of biomass and clean fuel exposure on pulmonary function during cooking among rural women in Tamilnadu, India

**DOI:** 10.6026/97320630017593

**Published:** 2021-05-31

**Authors:** Sarojini K Rajinikanth, M Chitra, N Kannan, Vinitha Baskaran, Madhan Krishnan

**Affiliations:** 1 Department of physiology, Sri Balaji Vidyapeeth, Puducherry, India; 2 Department of physiology, Shri Sathya Sai Medical College and Research Institute, Chennai and Sri Balaji Vidyapeeth, Puducherry, Tamil Nadu, India; 3 Department of physiology, Adhiparasakthi Institute of Medical Sciences and Research, Melmaruvathur, Tamilnadu, India; 4 Sri Sai Krupa Hospital, Puducherry, India; 5 Department of Biochemistry, Saveetha Dental College and Hospitals, Saveetha Institute of Medical and Technical Sciences, Saveetha University, Chennai, Tamil Nadu, India

**Keywords:** Biomass Fuel, clean fuel, exposure index, pulmonary function test, restrictive, obstructive disease, spirometry

## Abstract

It is of interest to document data on the comparative analysis of biomass and clean fuel exposure on pulmonary function during cooking among rural women. The study consisted of 100 biomass and 100 LPG fuel using women with no smoking habits and other related illness Parameters such as FVC, FEV1, FEV1/FVC, PEFR, FEF25-75%were obtained using the computerized spirometry to assess the pulmonary function in these subjects. The collected data were analyzed using the Student t-test method and Pearson correlation. The exposure index for biomass fuel users is 69.78±27.25 showing high exposure duration during cooking. The parameters for pulmonary functions significantly declined in FVC (42.34±13.6), FEV1 (45.55±15.98), PEFR (34.11±14.78) and FEF25-75% (45.56±23.00) for biomass fuel user. However, this is not true for FEV1/FVC ratio (107.56±16.9). The increase in PFT suggests the restrictive and obstructive patterns of pulmonary diseases. There was a negative correlation between increased duration of cooking and the value of FEV1/FVC (r = -0.2961), FEF25-75% (r = -0.3519) and PEFR (r = -0.2868). Thus, the deformation of pulmonary function due to extended exposure of biomass fuel for cooking women in rural Tamilnadu is shown using parameter features such as high exposure index, overcrowded area and improper ventilated houses.

## Background

In the rural regions of developing countries, most people use biomass fuels such as wood, cow dung, crop residues etc. for cooking purpose [1]. People were exposed to air pollution due to smoke from industry and vehicle causing chronic obstructive pulmonary disease in urban areas [2,3]. The particulate matters (fine or ultra fine) in air vary in size, composition and origin [4]. The particulate material such as gaseous pollutants, organic pollutants and heavy metals etc. progressively changes the indoors environmental [5]. The inhaled pollutants directly enter into the respiratory system and reach the circulation casuing deleterious effect on various organs among women and children during cooking [6]. The biomass smoke causes COPD by pulmonary and systematic inflammation using pro-inflammatory agents such as TRP (Transient Potential Receptor) and TLR (Toll-like receptors) and genotoxic effect of oxidative stress [7]. Nowadays 'Biomass exposure index' is used clinically as a tool to analyze the risk of developing the disease and to identify the minimum threshold of exposure. Biomass exposure index' is often used to calculate the minimum threshold of exposure and it is calculated by the average hours spent on cooking per day multiplied by the number of years of cooking [8]. It is known that there is an increase in enormous uncovered health burden such as respiratory and non-respiratory illnesses among the biomass fuel users [9]. Therefore, it is of interest to document data on the comparative analysis of biomass and clean fuel exposure on pulmonary function during cooking among rural women.

## Subjects and Methods:

### Study design:

#### Selection and description of participants:

A comparative study between rural biomass fuel user and clean fuel user for cooking was conducted around the Kancheepuram district, Tamilnadu. India. Clinical evaluation was done before doing the Spirometric test. Age (18-55 yrs) and BMI matched healthy women using the biomass fuel group and clean fuel and duration of exposure to cooking for minimum of 5 yrs were included. History of any diseased condition and smoking for the biomass fuel and clean fuel group were excluded. Detailed information about Anthropometry data were collected for the both groups such as Age, Height, Weight and BMI. The ventilation profile or data such as House type, Number of rooms, Placement of kitchen, Type of kitchen, presence or absence of Ventilation, Type of biomass fuel, and Duration of cooking were also collected. Pulmonary Function Test was done for every individual with the help of SPIROMETRY [MODEL: Helios 401, Version: 3.1.85], [10] which is based on European Respiratory standards [11] and assess [12] the Parameters such as FVC (Forced Vital Capacity), FEV1 (Forced Expiratory Volume in one second), FEV1/FVC ratio, PEFR (Peak Expiratory Flow Rate) and FEF 25-75% were done for the biomass fuel group and clean fuel group.

#### Informed consent and ethnical approval:

Written consent was taken in the regional language before collecting the data from each individual for the study purpose. The Institutional Ethical committee approved the study.

#### Statistics:

The student t test used to differentiate the groups at 5% level of significance. The correlation between the duration of exposure and PFT in biomass groups was assessed using the spearman correlation coefficient with 5% level of significance.

**Figure 1:**
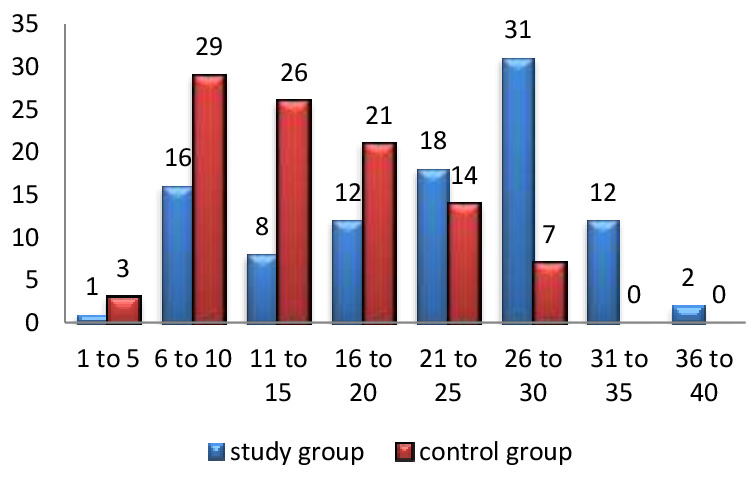
Frequency Distribution of Duration of Cooking in Years for Biomass Fuel Group and Clean Fuel Group.

Figure 1 clearly shows the details of the number of persons exposed to cooking in years for biomass fuel and clean fuel group.

**Figure 2:**
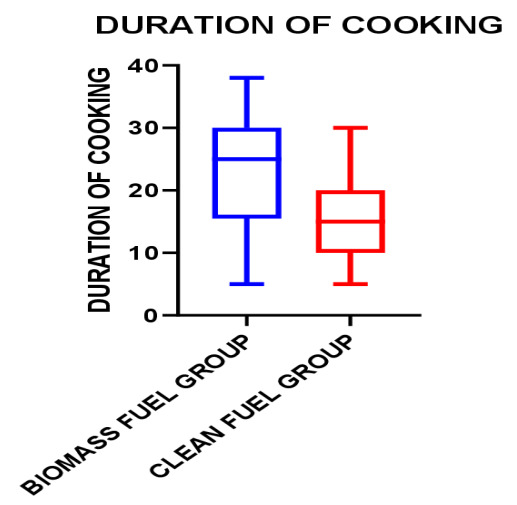
Box Plot Diagram for Exposure Index in Hours-Years for Biomass Fuel Group and Clean Fuel Group.

In Figure 2, box plot graph shows the higher median value for the biomass fuel group than the clean fuel group. The value between Q1 and Q3 for the biomass fuel group was higher than the clean fuel group.

## Results and Discussion:

The study was conducted on 200 women who were exposed to biomass fuel (n=100) and clean fuel (n=100). The pulmonary function test parameters were recorded and compared between the two groups. The groups were selected with an equal number of participants and the age between 18 - 55years. The rural population in India is still using the unprocessed biomass fuel for cooking in the indoor kitchen as well as an outdoor kitchen. The biomass fuel produces various products that alter the pulmonary functions among rural women. Previous studies have shown a significant decline in FVC, FEV1, and PEFR except FEV1/FVC ratio among biomass fuel exposure among those who used an indoor kitchen without a window for more than 20 years and the author showed the value of FVC was reduced more than that in FEV1 and FEV1/FVC ratio was normal, which indicated parenchymal restrictive lung disease [13]. Similarly in our study FVC, FEV1, PEFR and FEF25-75% were statistically reduced in biomass fuel users except FEV1/FVC ratio. FEF 25-75% is a more sensitive indicator and used as a potential diagnostic tool for the early detection of small airway function. The particulate matter (PM2.5µm) can easily penetrate the lungs, which affects the small airway and lung parenchyma [14,15]. Similarly, in our study, there was a significant decline in FEF25-75%, which indicates early small airway obstruction. A study reports showed a significant decline in PEFR with increasing duration of exposure due to chronic exposure to biomass fumes in poor ventilation of indoor kitchen which causes inflammation of airways among women [16]. Similarly, we also observed in our study a significant decline in PEFR among rural women who were prolonged exposure to biomass smoke, which indicates large airway obstruction.

Earlier a study showed a decline in FVC, FEV1, FEV1/FVC, FEF25-75% and PEFR caused by irritant gases and particulate matter released by biomass fuel combustion due to hypertrophy of mucosal cells which reflects the deficit in small and large airway function and lung parenchyma [17,18]. Chronic exposure to biomass smoke may cause an inflammatory reaction in the lungs, as a result of obstructive lung disease and shows some radiological signs of restrictive lung disease such as fibrotic bands, nodular opacities, and perivascular thickening [19,20]. Similarly, our result was showing a significantly decline in FVC, FEV1, FEF25-75%, PEFR and reduced value in FEV1/FVC which indicate the pattern of both obstructive and restrictive lung disease. Previous studies showed significant decline in FVC, FEV1, FEV1/FVC, FEF25-7% and PEFR with high biomass exposure index for biomass fuel group, which indicates the obstructive pulmonary disease due to chronic exposure of high concentration of the irritable substance and high biomass index as well as scarce ventilation [21,22, 23]. The minimum threshold of biomass exposure index is 60, which is significant risk to develop chronic bronchitis among women, [24] Similarly, our results show the exposure index (69.78± 27.25) which was higher in biomass fuel than clean fuel with a decline in FVC, FEV1, FEF25-7% and PEFR except for FEV1/FVC ratio. The exposure index based on the hours per day and years exposed to the biomass smoke. In a study showed the association between the spending hours per day and for more the 15 years exposure to biomass smoke which cause the adverse respiratory symptom [25]. Similarly our result showed the high exposure index which cause chronic bronchitis among rural women. A negative correlation was observed between lung function parameter (observed PEFR) with exposure index (r = –0.51). This indicates the affect of large airways obstruction caused by irritant gases and particulate matter, which induce hypertrophy of mucosal cells [26]. Similarly in our study shows negative correlation between duration of cooking with pulmonary function parameter of FEF 25-75% (r = -0.3519; p= 0.0003) and PEFR(r = -0.2868; p = 0.0038) for biomass fuel users. It indicates the obstruction in small airways and large airways. Another study showed the negative correlation of FVC, FEV1, FEV1/FVC with the duration of exposure in years, [27] similarly our study shows also exhibited a negative correlation of FEV1/FVC (r = -0.2961; p=0.0028). The effect of quality of life due to chronic biomass fuel exposure and poor ventilation as indicated by the higher exposure index is shown in the data.

## Conclusion:

Data shows the deformation of pulmonary function due to extented exposure of biomass fuel for cooking women in rural Tamilnadu is shown using parameter features such as high exposure index, overcrowded area and improper ventilated houses.
